# Feasibility of Anti-CEA Dye Conjugate for Cancer-Specific Imaging in Gastric Cancer Cell Lines and Mouse Xenograft Models

**DOI:** 10.3390/cancers17172937

**Published:** 2025-09-08

**Authors:** Kyoungyun Jeong, Annie Eunhee Koo, Jaeun Yoo, Ji-Yeon Shin, Leena Lim, Hyun Myong Kim, Ji-Yong Park, Yun-Sang Lee, Yoon-Jin Kwak, Hye Seung Lee, Yie-Ri Yoo, Bérénice Framery, Karen Dumas, Françoise Cailler, André Pèlegrin, Do-Joong Park, Han-Kwang Yang, Seong-Ho Kong, Hyuk-Joon Lee

**Affiliations:** 1 Cancer Research Institute, College of Medicine, Seoul National University, 101 Daehak-ro, Jongro-gu, Seoul 03080, Republic of Korea; kyjeong1210@snu.ac.kr (K.J.); 999eunhee@gmail.com (A.E.K.);; 2 Department of Nuclear Medicine, Seoul National University Hospital, 101 Daehak-ro, Jongro-gu, Seoul 03080, Republic of Korea; 3 Department of Pathology, Seoul National University Hospital, Seoul National University College of Medicine, 101 Daehak-ro, Jongro-gu, Seoul 03080, Republic of Korea; 4 SurgiMAb, 10 Parc Club du Millénaire, 1025 Avenue Henri Becquerel, 34000 Montpellier, France; 5 IRCM, Université de Montpellier, Inserm, ICM, 208 Rue des Apothicaires, 34298 Montpellier CEDEX 5, France; 6 Department of Surgery, Seoul National University Hospital, 101 Daehak-ro, Jongro-gu, Seoul 03080, Republic of Korea; 7 VITCAL, Co., Ltd., 101 Daehak-ro, Jongro-gu, Seoul 03080, Republic of Korea

**Keywords:** carcinoembryonic antigen, fluorescence, stomach neoplasms, immunoconjugates, heterografts, laparoscopy

## Abstract

**Purpose:** Near-infrared fluorescence-guided surgery (FGS) using cancer-specific tracers is promising for tailored gastric cancer (GC) surgery. Carcinoembryonic antigen (CEA) is a potential target due to its high expression in various digestive cancers, including GC. **Materials and Methods:** SGM-101, a chimeric anti-CEA monoclonal antibody conjugated with the near-infrared dye BM-104, was evaluated in GC. CEA expression was identified in GC cell lines at the mRNA and protein levels. Xenograft models (MKN-45, SNU-16, SNU-668, 85As2mLuc) were established in mice and injected with SGM-101 or PBS. Biodistribution was monitored using in vivo fluorescence imaging. Tumors were further analyzed by immunofluorescence. In a peritoneal carcinomatosis model, 85As2mLuc cells were injected intraperitoneally, and tumors were evaluated by bioluminescence and fluorescence and histology. **Results:** MKN-45, SNU-16, and 85As2mLuc were CEA-positive, while SNU-668 was CEA-negative. Flow cytometry confirmed CEA expression: MKN-45 (98%), SNU-16 (85.6%), SNU-668 (6.42%) and 85As2mLuc (78.4%). SGM-101 selectively targeted CEA-expressing tumors, with fluorescence peaking at 48 h, and immunofluorescence verified localization in tumor cells. In the peritoneal models, SGM-101 enabled precise detection of CEA-positive tumors. **Conclusions:** This study provides the first evidence for the feasibility of SGM-101 in gastric cancer, demonstrating its novelty and translational potential as a cancer-specific imaging agent for fluorescence-guided surgery.

## Simple Summary

Surgical removal remains essential for the treatment of gastric cancer, yet it is often difficult to accurately identify tumor boundaries or metastatic lesions during the operation. Indocyanine green, the current imaging agent, lacks cancer specificity to a cancer-related protein called carcinoembryonic antigen. By attaching a fluorescent dye to this antibody, SGM-101 enables surgeons to visually distinguish cancerous tissue from normal tissue in real time. We confirmed that the signal intensity of SGM-101 correlates with cancer-specific protein expression, allowing precise visualization of both primary tumors and peritoneal metastasis. Importantly, this is first study to demonstrate the feasibility and innovation of applying SGM-101 in gastric cancer, highlighting its potential to develop from preclinical studies to clinical application. This approach may improve the detection of hidden lesions, and support personalized surgery. Our findings suggest that SGM-101 has strong potential for use in fluorescence-guided surgery for patients with gastric cancer.

## 1. Introduction

Gastric cancer (GC) remains a major global health burden, with over one million new cases and an estimated 700,000 deaths annually [1,2,3,4]. Despite advances in systemic therapy, curative treatment largely depends on complete surgical resection. However, visual identification of tumor margins and lymph node metastasis during surgery remains challenging, often resulting in radical resections that carry risks of complications and reduced quality of life [5,6].

In early-stage GC, endoscopic resection is preferred for patients without nodal involvement, but advanced tumors require gastrectomy and lymphadenectomy [7,8,9]. Due to the complexity of gastric lymphatic drainage, suitable tracers that can guide accurate lymph node mapping while ensuring oncologic safety are increasingly needed. Near-infrared (NIR) fluorescence-guided surgery (FGS) has emerged as a promising tool for tumor detection, offering high sensitivity and moderate tissue penetration [10,11,12,13,14,15,16,17]. Among currently used tracers, indocyanine green (ICG) is most widely applied; however, its lack of tumor specificity limits its potential in achieving precise resection [18,19,20,21,22,23]. In this context, antibody-based imaging agents targeting tumor-associated antigens may enhance surgical accuracy [24,25,26,27,28].

Carcinoembryonic antigen (CEA), expressed in 34–66% of GCs with minimal expression in normal gastric tissue, represents a strong candidate for targeting imaging [29,30,31,32,33,34,35,36,37,38,39,40]. SGM-101 (Surgimab, Montpellier, France) is a chimeric monoclonal antibody conjugated to an NIR dye, which has shown cancer-specific imaging in colorectal and pancreatic cancers [24,25,26,28,41,42,43,44]. However, its use in GC has not been fully characterized. In this study, we demonstrated for the first time the feasibility and innovation of applying SGM-101 in GC. SGM-101 bound specifically to GC cell lines in vitro and enabled real-time detection in two in vivo models. Notably, the peritoneal model revealed reliable CEA detection even at small tumor burdens, highlighting the novelty of this study and supporting the SGM-101 to visualize disseminated tumors. These results support the potential of SGM-101 as a promising tool for fluorescence-guided surgery in GC patients in the future.

## 2. Materials and Methods

### 2.1. Preparation of Fluorescent Dye-Labeled Anti-CEA Antibody; SGM-101 and Isotype Control-101

**Figure 1:**
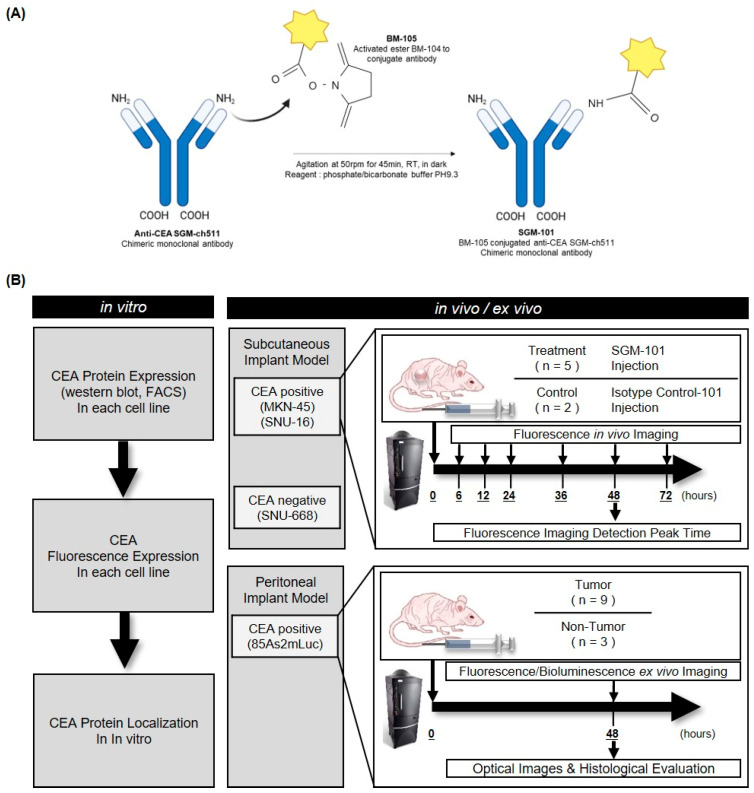
Preparation of SGM-101, cell lines, and animal models for fluorescence imaging. Study scheme: in vitro, in vivo, and ex vivo experiments were conducted. (**A**) Preparation of SGM-101. (**B**) Three cell lines were implanted into the subcutaneous layer of the right flank of BALB/c-nu mice. Fluorescence imaging was obtained using the In Vivo Imaging System at multiple time points after systemic administration of SGM-101. In the peritoneal seeding model with the 85As2mLuc cell line, mice were autopsied 48 h after injection of SGM-101, and the fluorescence imaging results of suspected nodules were compared with bioluminescence imaging and pathological confirmation.

A chimeric monoclonal antibody, SGM-101, was synthesized by conjugating the anti-CEA monoclonal antibody SGM-ch511 with the near-infrared fluorescent dye BM-105. The conjugation was achieved via an activated ester of BM-105 reacting with amino groups (-NH_2_) of the antibody, and the synthesis process and characterization of SGM-101 have been previously described [45]. (Figure 1A) A polyclonal immunoglobulin (IgG) isotype control from BioXcell (BioXcell, Lebanon, NH, USA) conjugated with BM-105 was used as a control group and named ‘Isotype Control-101′. The dye-to-antibody molar ratio was consistently maintained at approximately 1–2 [45], calculated using the molarities of the antibody and dye determined from optical density (OD) readings. Absorbance was measured at 280 nm and 685 nm using a spectrophotometer (Thermo Fisher Scientific, Waltham, MA, USA). For the FACS, the anti-CEA mAb (Santa Cruz Biotechnology, Dallas, TX, USA) conjugated with Alexa Fluor-680 (Thermo Fisher Scientific, Waltham, MA, USA), named ‘SC-680′, was used as a positive control to detect CEA-positive cells.

### 2.2. Cell Culture

The MKN-45 (American Type Culture Collection, Manassas, VA, USA), SNU-16, and SNU-668 cell lines (Korean Cell Line Bank, Seoul, Republic of Korea) were selected for subcutaneous models as representative gastric cancer cell lines with different levels of CEA RNA expression (positive and negative). A genetically modified gastric cancer cell line 85As2mLuc, which expresses CEA and emits bioluminescence upon administration of D-luciferin, was generously provided by Prof. Yanagihara K at the National Cancer Center in Japan for establishing the peritoneal seeding model [46]. All cell lines were cultured in RPMI1640 (Welgene, Gyeongsan, Republic of Korea), supplemented with 10% (*v*/*v*) fetal bovine serum (Gibco, Thermofisher Scientific, Waltham, MA, USA) and 1% penicillin/streptomycin (Gibco, Thermofisher Scientific, MA, USA). They were incubated at 37 °C in a 5% CO_2_ humidified environment.

### 2.3. Western Blotting

Protein lysates were obtained with a mixture of RIPA buffer and phosphatase and protease inhibitor cocktails (Thermofisher Scientific, MA, USA), and the protein concentration was measured by BCA assay (Thermofisher Scientific, MA, USA). The lysates containing protein were separated by SDS-PAGE (Thermofisher Scientific, MA, USA). The membrane was probed with primary antibodies against GAPDH (Abcam, Cambridge, Cambs, UK) and CEA (Santa Cruz Biotechnology, Dallas, TX, USA) with HRP-conjugated secondary antibodies: mouse IgG (Cell Signaling Technology, Danvers, MA, USA) and rabbit IgG (Cell Signaling Technology, Danvers, MA, USA). This was visualized with Enhanced Chemiluminescent Horseradish Peroxidase substrate (Thermofisher Scientific, MA, USA) and imaged by ChemiDoc (Bio-Rad, Hercules, CA, USA).

### 2.4. Flow Cytometry

A total of 1 × 10^6^ cells were collected into a test tube and centrifuged at 3000 rpm for 5 min at 4 °C. The cells were incubated for 30 min with 10% (*v*/*v*) fetal bovine serum (Thermo Fisher Scientific, MA, USA) to minimize non-specific binding. Subsequently, they were treated with a primary antibody conjugated to a fluorescent dye, SGM-101, Isotype Control-101, SC-680, as well as the fluorescent dye BM-105 for 1 h at 4 °C. Propidium Iodide (Thermofisher Scientific, MA, USA) was added to each test tube for the live and dead cell sorting. FACS Canto II (BD Bioscience, San Jose, CA, USA) was for fluorescence signal analysis. The data were analyzed and visualized using FlowJo (BD Bioscience, San Jose, CA, USA).

### 2.5. Immunocytochemistry

1 × 10^5^ cells were seeded onto poly-L-lysine (Sigma-Aldrich, St. Louis, MO, USA) coated coverslips in a 6-well dish. At 80% confluency, they were fixed with 4% paraformaldehyde solution (Thermofisher Scientific, MA, USA) and incubated overnight at 4 °C with both SGM-101 and Isotype-101 conjugates. Fluorescence detection of CEA was performed using STELLARIS (Leica Microsystems, Wetzlar, HE, Germany), and quantification was performed by measuring the signal intensity of fluorescence in five randomly acquired images using the LAS X program (Leica Microsystems, Wetzlar, HE, Germany). DAPI intensity was used for normalization.

### 2.6. Mouse Modeling

Subcutaneous xenograft models were established by injecting 1 × 10^7^ cells from each cell line (MKN-45, SNU-16, and SNU-668) into the right flank of 6-week-old female BALB/c-nu mice. Tumor growth was monitored twice weekly by measuring tumor size with caliper, and the volume was calculated with the formula: 1/2 × length × width^2^ (mm^3^). For the peritoneal seeding model, 2 × 10^6^ cells from the 85As2mLuc cell line, which emit bioluminescence light due to luciferase activity, were intraperitoneally injected into 6-week-old female BALB/c-nu mice. The tumor growth in the peritoneal cavity was measured using an IVIS Spectrum System (Perkin Elmer, Waltham, MA, USA) based on bioluminescence activity.

### 2.7. Fluorescence Tumor Detection Imaging

SGM-101 was administered intravenously to mice via the tail vein at a dosage of 2.5 mg/kg. Fluorescence imaging was performed using an IVIS Spectrum with excitation at 680 nm and emission at the peak wavelength of 720 nm. The fluorescence signal intensity of Region of Interest (ROI) was quantified by the radiant efficiency ([p/s/cm^2^/sr]/[μW/cm^2^]) using Living Image software 2.50.1 (Perkin Elmer, Waltham, MA, USA) (Figure 1B). For the subcutaneous model, imaging was conducted at 6, 12, 24, 48, and 72 h after injection to understand the biodistribution for 5 mice. A total of 2 mice for each cell line were autopsied at 48 h after injection, and the tumor and internal organs were imaged for their fluorescence signal. For the peritoneal seeding model, establishment of peritoneal seeding nodules was checked by luminescence imaging using IVIS system 10 min after intraperitoneally injected D-luciferin (Promega, Madison, WI, USA). After laparotomy, visually suspected seeding nodules were collected and imaged by fluorescence and luminescence imaging, and then evaluated histologically, such as H&E.

### 2.8. Histologic Evaluation

For the ex vivo Fluorescence Imaging, the dissected tissues were mounted in OCT embedding compounds (Sakura Finetek, Torrance, CA, USA) and frozen at −80 °C. Cryosections were conducted in 4 µm-thick tissue and mounted with DAPI. These were imaged with STELLARIS to scan whole-size samples to see the micro-distribution. For the Immunohistochemistry, primary tumors, which were excised from mice, were fixed in 10% neutral buffered formalin solution (Sigma-Aldrich, St. Louis, MO, USA) and consecutively paraffin-embedded. For histological analysis, the formalin-fixed paraffin-embedded (FFPE) tissues were sectioned at a thickness of about 4 μm, stained with hematoxylin and eosin (H&E) using Image Scope (Aperio, Leica Biosystems, Vista, CA, USA). Immunohistochemistry (IHC) staining of 4 μm slides was performed using CEA antibody (Abcam, Cambridge, Cambs, UK). The staining was performed with Bond-Max immunostainner, and bond polymer refine detection kit (Leica Microsystems, Wetzlar, HE, Germany) according to the manufacturer’s instructions.

### 2.9. Statistical Analysis

Wilcoxon signed-rank test and Mann–Whitney test were performed using GraphPad PRISM version 9.0 (GraphPad Software Inc., Boston, MA, USA) for non-parametric data. All parametric data were presented as means ± standard deviation (SD). Statistical significance in biodistribution was defined as Kruskal–Wallis one-way analysis of variance.

## 3. Results

### 3.1. Different CEA Fluorescence Detection by SGM-101 at the Cell Surface of Each GC Cell Line

**Figure 2:**
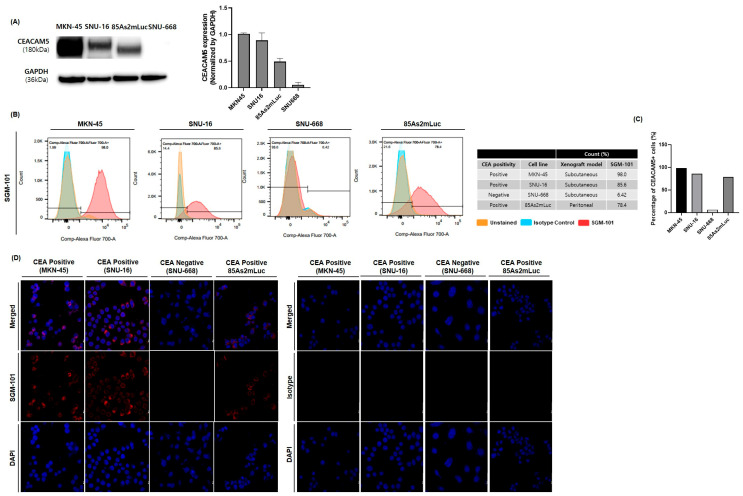
CEA expression and detection pattern in gastric cancer cell lines. (**A**) Western blotting analysis of Carcinoembryonic Antigen protein expression in cell lines. (**B**) Flow cytometry analysis demonstrated Carcinoembryonic Antigen-dependent binding of SGM-101 to each cell line, while isotype control conjugated with BM-105 showed minimal binding, similar to unstained control. (**C**) The positivity rate of Carcinoembryonic Antigen varied across different cell lines. MKN45 exhibited the highest positivity rate at 98.6%, followed by SNU16 at 85.6%, 85As2mLuc at 78.4%, and SNU-668 with the lowest rate at 6.42%. These rates correlate with Carcinoembryonic Antigen expression levels, as confirmed by Western blot analysis. (**D**) Confocal microscopy images confirmed the attachment of SGM-101 to the Carcinoembryonic Antigen molecule on the surface of the cell lines. In contrast, the isotype control did not show attachment of Carcinoembryonic Antigen. The uncropped blots are shown in Figure S2.

The Western blot results show that CEA expression levels in MKN-45 and SNU-16, except in SNU-668. CEA expression was also observed in the 85As2mLuc cell line (Figure 2A). The binding levels of SGM-101 to the CEA and the relative fluorescence intensity in each cell line were confirmed by FACS analysis. In each case, the results were normalized to the binding of an isotype control labeled with the relevant dye, BM-105 and Isotype Control-101. The fluorescence level of each cell line indicated the specificity of SGM-101 for CEA (Figure 2B,C). The confocal microscopy results also demonstrated that SGM-101 detects CEA on the surface of each cell line with binding specificities. The fluorescence intensity of SGM-101, which was normalized with DAPI intensity and cell numbers, showed CEA expression-dependent manners. In contrast, the use of isotype control images showed no detectable CEA, which is consistent with previous findings (Figure 2D).

### 3.2. SGM-101 Detection in Subcutaneous Gastric Tumors According to Their CEA Expression and in a Time-Dependent Manner

**Figure 3:**
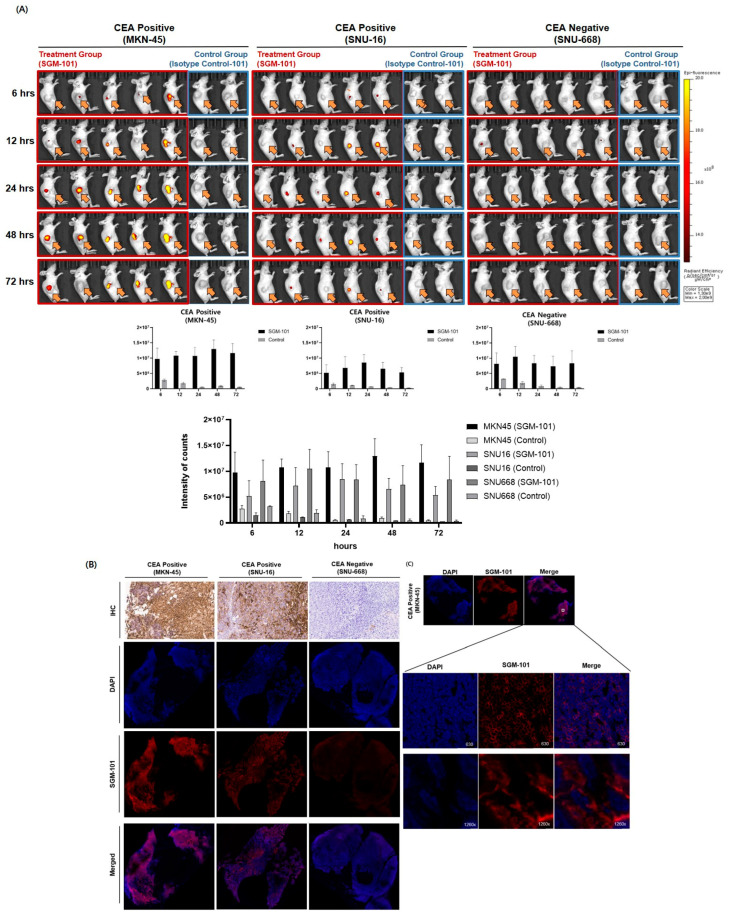
Subcutaneous xenograft model imaging: (**A**) Imaging at various time points was performed after intravenous injection of SGM-101 (experimental group) and phosphate-buffered saline (control) into the tail veins of mice (*n* = 5, *n* = 2, respectively). The imaging results were categorized into three groups based on Carcinoembryonic Antigen positivity. The Carcinoembryonic Antigen-positive group showed the best detection rates at 48 h post SGM-101 injection, while negative group exhibited a 0% detection rate at all time points. The arrows indicate the location of the tumor. (**B**) Following in vivo imaging system imaging, the tumors were excised and analyzed ex vivo for Carcinoembryonic Antigen expression using Immunohistochemistry. The results showed a direct correlation between tumor Carcinoembryonic Antigen expression and Carcinoembryonic Antigen positivity, demonstrating that SGM-101 effectively targets Carcinoembryonic Antigen-positive cancer cells. A section of the ex vivo tumor tissue was cryosectioned to assess microdistribution. The analysis revealed a correlation between Carcinoembryonic Antigen expression and SGM-101 intensity. (**C**) In the MKN45 cells, which showed the highest Carcinoembryonic Antigen expression, a strong SGM-101 fluorescence signal was detected on the cell surface when viewed under magnification.

We conducted in vivo imaging using a mouse xenograft model with gastric cancer cell lines exhibiting different levels of CEA expression. The cell lines were subcutaneously injected into mice, and fluorescent images were captured using SGM-101 and isotype IgG. (SGM-101 and isotype IgG were administered to five mice and two mice, respectively.) After injecting SGM-101 and isotype IgG via the tail vein, imaging was performed at 6, 12, 24, 48, and 72 h. The fluorescence intensity was correlated with CEA expression. Based on saturated intensity, CEA-positive (MKN-45) mice showed detection rates of 20% at 6 h, 40% at 12 h, 80% at 24 h, 100% at 48 h, and 60% at 72 h (*p* = 0.004). CEA-positive (SNU-16) mice had detection rates of 0% at 6 h, 40% at 12 h, and 20% from 24 to 72 h (*p* = 0.0011), while CEA-negative (SNU-668) mice had a 0% detection rate from 6 to 72 h (*p* = 0.0003) (Figure 3A). Tumor samples obtained after imaging were analyzed for CEA protein expression using IHC (Figure 3B). Additionally, IF imaging using FFPE slides showed that CEA expression was similarly proportional to fluorescence intensity (Figure 3B,C). When we magnified the slide results for the MKN45 cell line, we observed strong positivity around the stained nuclei, indicating that CEA is a surface glycoprotein and that SGM-101 effectively targets CEA (Figure 3C). Therefore, based on the data, we confirmed that SGM-101 is an effective probe for targeting gastric cancer cells with high CEA expression. Moreover, the highest detection rate observed at 48 h after intravenous administration of SGM-101 suggests that this antibody mimetic has the potential to more accurately target CEA protein after sufficient circulation within the body.

**Figure 4:**
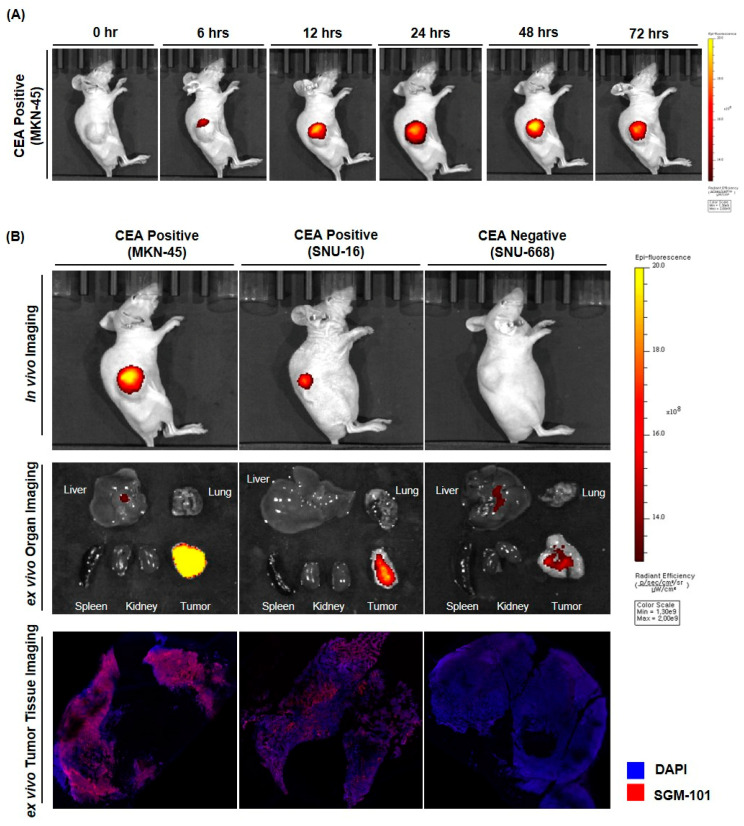
Biodistribution of SGM-101 in subcutaneous xenograft model: (**A**) Images of mice at 6, 12, 24, 48, 72 h after injection with SGM-101. It shows that distribution of SGM-101 in gastric cancer xenografts. A total of 48 h is the highest accumulation in cancer area. (**B**) Images of mice, organ ex vivo, tumor tissue ex vivo at 48 h after injection with SGM-101. With images of mice, fluorescence signals and Carcinoembryonic Antigen expression show relationship to each other. With ex vivo images of organ, SGM-101 has high specificity for cancer. With ex vivo images of tumor, SGM-101 is attached to cell membranes.

The biodistribution of SGM-101 was analyzed in subcutaneous (SC) models from the high-CEA expressing cell line, MKN45. The highest signal intensity was observed 48 h after SGM-101 i.v. injection among the time points tested (0, 6, 12, 24, 48, and 72 h) (Figure 4A). CEA-specific fluorescent imaging in the three cell lines showed that the tumor fluorescence signal appeared in MKN-45 and SNU-16 in a CEA expression-dependent manner at 48 h after SGM-101 administration. In contrast, the fluorescent signal was barely detectable in SNU-668, where CEA expression was low. We also performed in vivo fluorescence imaging of representative mice for the three cell lines 48 h after the administration of SGM-101, followed by ex vivo fluorescence imaging after necropsy (Figure 4B). The intratumoral fluorescence brightness observed in both the in vivo imaging and the necropsy correlated with the CEA expression level in tumor tissue, as observed through immunofluorescence imaging and IHC. Additionally, 48 h after administration, residual fluorescence signals of SGM-101 were barely detectable in other organs, including the liver, spleen, and kidneys (Figure 4B and Figure S1). The text continues here (Figure 2).

### 3.3. The Bioluminescence and Fluorescence Tumor Imaging in Peritoneal Seeding Model

**Figure 5:**
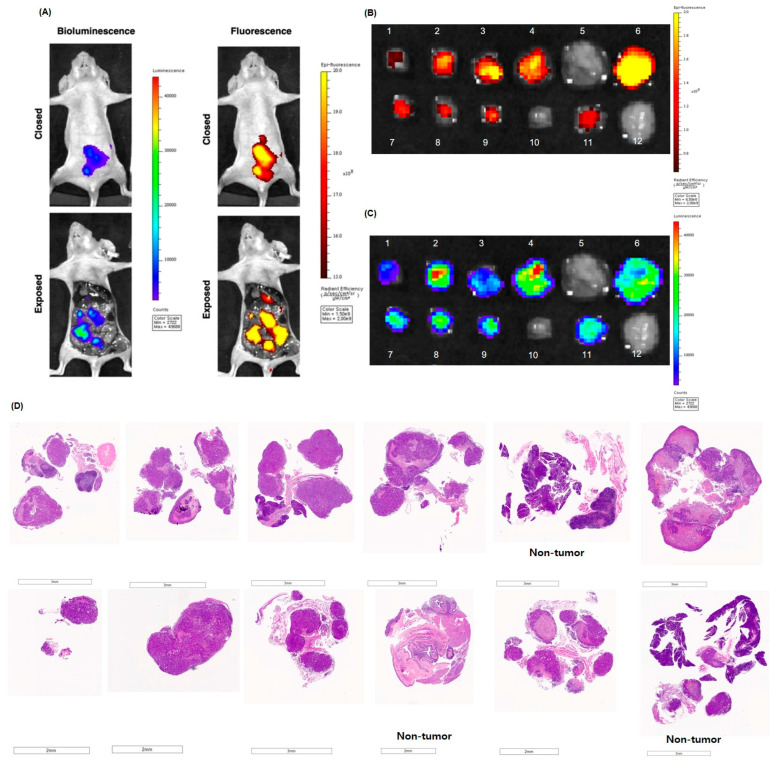
Ex vivo tumor fluorescence and bioluminescence imaging and histological evaluation in peritoneal seeding model. (**A**) The fluorescence signal generated by SGM-101 and the bioluminescence signal from the 85As2mLuc cell correlated well with each other in in vivo imaging. In the suspected seeding nodules, (**B**) Fluorescence signal, (**C**) the bioluminescence signal, and (**D**) histopathological evaluation are well correlated with each other.

Mice with an established peritoneal seeding model, induced by injection of the 85As2mLuc cell line into the peritoneal cavity, were intravenously administered SGM-101. After 48 h, D-luciferin was additionally injected into the peritoneal cavity, followed by autopsy and laparotomy. Nodules suspected of peritoneal seeding by visual inspection were harvested, and the fluorescent signal of CEA induced by SGM-101 was compared with the bioluminescent signal generated by the cell lines themselves through their luciferase activity (Figure 5A–C).

The brightness of the fluorescence observed in both in vivo imaging and autopsy correlated with the level of CEA expression in the tumor tissue, as confirmed by immunofluorescence imaging and IHC. At 48 h after injection, significant fluorescence signals from SGM-101 were nearly undetectable in non-tumor tissues, including the liver, spleen, and kidneys. Several peritoneal nodules that were visually suspected to be metastatic seeding did not exhibit fluorescence signals when examined with SGM-101. Upon further pathological examination, the nodules initially thought to represent peritoneal seeding, including those labeled as 5 (pancreas and lymph node), 10 (liver and intestinal wall), and 12 (pancreas), were confirmed to be normal tissues without any evidence of malignancy. Among the 12 nodules examined, 9 were confirmed as gastric cancer tissues, while 3 showed no cancerous cells. Importantly, no dysplastic or hyperplastic changes were identified in these nodules (Figure 5D).

The presence and intensity of fluorescent signals were correlated with those of bioluminescent signals, and were consistent with the presence of gastric cancer confirmed by a pathologist using H&E staining.

## 4. Discussion

ICG-based near-infrared fluorescence imaging is recognized as a promising technique with the potential to guide both sentinel node navigation and standard lymph node dissection, leading to increased research and clinical implementation. However, the use of ICG has limitations, as it can rapidly and extensively spread and there may be differences in the speed and range of diffusion among individual patients, thereby hindering the full realization of patient-tailored surgery [10,20,21,47,48]. The development of cancer-specific tracers is expected to overcome or substitute for the limitations of ICG. Moreover, it could facilitate the visualization of cancer margins that ICG cannot detect, and enable prompt evaluation of peritoneal seeding, which is often visually challenging and has important clinical significance in GC. Additionally, the development of tumor-specific probes could be extended to confirm drug sensitivity in PDX models as well as allow for the assessment of surgical feasibility through imaging before surgery. Intraoperative frozen biopsy to assess the presence of cancer cells in the resection margin or suspected seeding nodules may not be available at some hospitals, and even where it is available, it could lead to a significant surgical delay of at least 30 min. Fluorescence imaging with cancer-specific tracers is expected to reduce such delays. The selection of a suitable target is a critical and challenging aspect of developing cancer-specific tracers. In order to expand their application from imaging to theranostics with treatment efficacy, it may be preferable to select therapeutic targets [49,50,51,52]. However, finding a target that is universally expressed in cancer cells is difficult. Even HER2, which is one of the most commonly expressed therapeutic targets for gastric cancer, is expressed in less than 20% of cases. On the other hand, while the usefulness of CEA as a therapeutic target has not been established, it is known to be expressed in various gastrointestinal malignancies and is recognized as one of the most promising candidates for fluorescence imaging [43,53]. The expression of CEA is known to be more than 90% in cases of colorectal cancer [54,55], whereas its expression in gastric cancer is not less defined. CEA is reported to be expressed in over 30% of cases [29], but at a lower frequency compared to colorectal cancer. However, a study by Jang et al. reported that 66.1% of gastric cancer cases were CEACAM5-positive [29], indicating that CEA may be expressed in a significant number of cases of gastric cancer as well.

The SGM-101 utilized in this experiment is an IgG monoclonal antibody that targets CEA and is conjugated with the fluorescent dye BM-105 [45]. Its physical characteristics have been described previously [26]. The conjugate exhibits high stability in human plasma, with no significant release of BM-104 dye from the antibody under physiological conditions. Pharmacokinetic analyses in rodent and canine models revealed a biphasic elimination profile typical of IgG1 antibodies, with dose-proportional plasma exposure and no evidence of unexpected accumulation. Toxicology studies also showed a favorable safety profile, with no observable adverse effect levels (NOAEL) of 40 mg/kg in rats and 5 mg/kg in dogs, corresponding to a more than ten-fold safety margin compared with the anticipated clinical dose. In addition, animal studies have shown its cancer-specificity in colon and pancreatic cancer [26,41], and multi-institutional clinical trials demonstrated its potential clinical utility in human surgery [24,25,44,53]. Consistent with these findings, our study using a gastric cancer xenograft model demonstrated the feasibility of applying SGM-101 for cancer-specific intraoperative imaging in gastric cancer.

SGM-101 demonstrated specific binding to CEA in the gastric cancer xenograft model, exhibiting a selective fluorescence signal that depended on the level of CEA expression. Consistent with previous preclinical studies, the highest signal intensity was detected 48 h post-injection of SGM-101 (Figure 3A). This duration corresponds to the pharmacokinetic properties of tracers based on IgG, which typically have relatively large molecular weights. Theoretically, smaller molecules such as Fab or affibody that penetrate tissue targets more quickly and exhibit rapid clearance from non-target tissues may provide advantages for clinical applications. However, developing small molecules that maintain high affinity while being optimized for the surgical time window presents a significant challenge. Often, achieving optimal efficacy requires larger injection volumes or modifications to extend the circulation time due to rapid clearance before reaching the target. Specifically, we observed a discrepancy between the IVIS imaging and IHC results after intravenously injecting SGM-101 into a tumor mouse model with low CEA expression. In particular, while the IVIS showed some intensity in the tumor area, the IHC results were completely negative. This discrepancy could be attributed to several factors.

First, it is important to note that the IHC used a commercially available antibody specific to CEA, whereas IVIS imaging utilized our newly developed probe, which may have different affinities for the protein. Second, autofluorescence signals from collagen-rich or calcified structures can be observed under 700 nm near-infrared (NIR) light, potentially contributing to weak background fluorescence in IVIS imaging [56,57,58]. Additionally, while the probe was administered via the bloodstream, it may not have accumulated sufficiently within the tumor to generate a strong IVIS signal. In contrast, the minimal CEA expression in the tumor tissue could have resulted in a negative IHC result, highlighting a potential discrepancy between the two detection methods. Third, IVIS imaging is highly sensitive and capable of detecting weak fluorescence signals, including residual probe fluorescence in the bloodstream or surrounding tissues. This residual signal may falsely appear to originate from the tumor, even in the absence of significant probe accumulation within the tumor itself.

Although SGM-101 generally demonstrates high specificity, low levels of nonspecific binding or background fluorescence could contribute to the weak signals observed in IVIS but not in IHC.

Nonetheless, the 48 h preoperative interval required for IgG-based tracers is not considered a significant clinical limitation.

SGM-101 was also evaluated in a peritoneal seeding model to explore its potential for rapid intraoperative detection of malignant nodules, which is a highly demanded medical need. In this model, luminescent cell lines (85As2mLuc) were employed to non-invasively confirm the establishment and growth of cancer cells in the peritoneal cavity, making them ready for fluorescence imaging. Luminescence imaging was conducted using a wavelength of approximately 560 nm, which did not interfere with the fluorescence signal of SGM-101 at 720 nm. The fluorescent signal of SGM-101 exhibited a strong correlation, both in location and intensity, with the luminescence signal of the cancer cells themselves as well as with the histological evaluation result.

The expression of CEACAM5 plays a critical role in both gastric adenoma and early gastric cancer, with a positivity rate of approximately 34–66% in early-stage gastric adenocarcinomas [29,30,36,59], suggesting its potential utility for early diagnosis. These findings underscore the importance of adopting multimodal diagnostic strategies, including the integration of imaging techniques, to enhance detection accuracy and reduce the likelihood of false-positive/negative results during endoscopic examinations. Nonetheless, CEA expression in gastric cancer is heterogeneous, varying across tumor subtypes, disease stages, and even within individual tumors [60,61,62]. Such heterogeneity may limit the reliability of CEA as a gastric cancer-specific marker and influence the sensitivity and specificity of CEA-targeted approaches. However, given the absence of a single definitive biomarker for gastric cancer, continued investigation of CEA-targeted strategies remains meaningful, with the hope of achieving successful clinical application.

This study has some limitations. First, the CEA expression levels in the cell lines used in the experiments were either high in two cases or almost absent in one case, since an intermediate CEA expression cell line could not be selected despite screening 37 cell lines. CEA expression in gastric cancer cell lines tends to be either high or almost absent. The CEA expression level that can produce clinically detectable fluorescence intensity needs to be validated through various cases in clinical trials. However, clinical results obtained in colorectal and pancreatic cancers make us confident in this approach [24,25,44,53].

The second limitation of this study is related to the imaging equipment used, the IVIS system, which was not a laparoscopy designed for human use. In Korea, all currently available near-infrared fluorescence laparoscopic systems are optimized for wavelengths around 820 nm for ICG, and equipment is not yet available. Although it remains to be confirmed whether the brightness observed on the IVIS system can be reproduced on a laparoscopic system, our previous study targeting HER2 detected a brightness equivalent to that observed in IVIS imaging using a laparoscopic system for 820 nm [11]. With the rapid advancements in fluorescence laparoscopic technology, it is anticipated that sensitive laparoscopic systems capable of displaying bright signals at 705 nm will become available soon. If a laparoscopic system that can detect both wavelengths becomes available, it may lead to the development of an efficient surgical approach that uses ICG at 820 nm to track the pathway of cancer cells and SGM-101 at 705 nm to confirm the presence of cancer in potential lesions.

The third limitation of this study is the lack of long-term evaluation of the biocompatibility of SGM-101. Although previous studies have demonstrated the safety of SGM-101, its biosafety may vary depending on the cancer type, injection dose, and method of administration. Therefore, further investigations will be necessary to clarify whether SGM-101 induces any potential effects in vivo.

In conclusion, SGM-101, a monoclonal antibody specific to CEA and emitting fluorescence at 705 nm, demonstrated binding and quantitative correlation with different levels of CEA expression in gastric cancer cell lines. In the subcutaneous xenograft model, the maximum fluorescent signal was observed 24–48 h after injection, and the signal intensity correlated with CEA expression. In the peritoneal seeding model, SGM-101 outperformed visual inspection in determining the presence of cancer cells within suspected nodules. These results underscore the robustness and translational potential of SGM-101 as a fluorescence-guided surgical probe. Looking forward, validation in clinical gastric cancer samples and assessment of its applicability across laparoscopic wavelengths will be crucial. Nevertheless, our findings strongly support the promise of SGM-101 as a powerful tool for cancer-specific near-infrared fluorescence intraoperative imaging and navigation in laparoscopic gastric cancer surgery.

## 5. Conclusions

This study demonstrates that SGM-101, a CEA-targeted fluorescent antibody, enables cancer-specific near-infrared imaging in gastric cancer models. Its signal intensity correlates with CEA expression and allows clear visualization of tumors and peritoneal lesions within a clinically applicable time frame. These findings suggest that SGM-101 may enhance surgical precision and help overcome the limitations of current non-specific tracers.

Further clinical validation is warranted to assess its effectiveness across diverse gastric cancer cases and its compatibility with laparoscopic systems.

## Data Availability

The raw data supporting the conclusions of this article will be made available by the authors on request.

## Supplementary Materials

The following supporting information can be downloaded at: https://www.mdpi.com/article/10.3390/cancers17172937/s1, Figure S1: Ex vivo tumor histological image; Figure S2: original images of western blot.

## Abbreviations

The following abbreviations are used in this manuscript:

GC	Gastric Cancer
CEA	Carcinoembryonic antigen
NIR	Near-infrared
FGS	Fluorescence-guided surgery
ICG	Indocyanine Green
O.D.	Optical Density
ROI	Region of Interest
FFPE	Formalin-fixed paraffin-embedded
H&E	Hematoxylin and eosin
SD	Standard Deviation
SC	Subcutaneous

## References

[1] Siegel R.L., Miller K.D., Fuchs H.E., Jemal A. (2022). Cancer statistics, 2022. CA Cancer J. Clin..

[2] Wong M.C.S., Huang J., Chan P.S.F., Choi P., Lao X.Q., Chan S.M., Teoh A., Liang P. (2021). Global Incidence and Mortality of Gastric Cancer, 1980–2018. JAMA Netw. Open.

[3] Sung H., Ferlay J., Siegel R.L., Laversanne M., Soerjomataram I., Jemal A., Bray F. (2021). Global cancer statistics 2020: GLOBOCAN estimates of incidence and mortality worldwide for 36 cancers in 185 countries. CA Cancer J. Clin..

[4] Ferlay J., Colombet M., Soerjomataram I., Parkin D.M., Piñeros M., Znaor A., Bray F. (2021). Cancer statistics for the year 2020: An overview. Int. J. Cancer.

[5] Kim T.H., Kim I.H., Kang S.J., Choi M., Kim B.H., Eom B.W., Kim B.J., Min B.H., Choi C.I., Shin C.M. (2023). Korean Practice Guidelines for Gastric Cancer 2022: An Evidence-based, Multidisciplinary Approach. J. Gastric Cancer.

[6] Park S.-H., Suh Y.-S., Kim T.-H., Choi Y.-H., Choi J.-H., Kong S.-H., Park D.J., Lee H.-J., Yang H.-K. (2021). Postoperative morbidity and quality of life between totally laparoscopic total gastrectomy and laparoscopy-assisted total gastrectomy: A propensity-score matched analysis. BMC Cancer.

[7] Lordick F., Carneiro F., Cascinu S., Fleitas T., Haustermans K., Piessen G., Vogel A., Smyth E. (2022). Gastric cancer: ESMO Clinical Practice Guideline for diagnosis, treatment and follow-up☆. Ann. Oncol..

[8] Joshi S.S., Badgwell B.D. (2021). Current treatment and recent progress in gastric cancer. CA Cancer J. Clin..

[9] Yoon J.Y., Shim C.N., Chung S.H., Park W., Chung H., Lee H., Shin S.K., Lee S.K., Lee Y.C., Park J.C. (2014). Impact of tumor location on clinical outcomes of gastric endoscopic submucosal dissection. World J. Gastroenterol. WJG.

[10] Park J.-H., Berlth F., Wang C., Wang S., Choi J.-H., Park S.-H., Suh Y.-S., Kong S.-H., Park D.J., Lee H.-J. (2022). Mapping of the perigastric lymphatic network using indocyanine green fluorescence imaging and tissue marking dye in clinically advanced gastric cancer. Eur. J. Surg. Oncol..

[11] Jeong K., Kong S.H., Bae S.W., Park C.R., Berlth F., Shin J.H., Lee Y.S., Youn H., Koo E., Suh Y.S. (2021). Evaluation of Near-infrared Fluorescence-conjugated Peptides for Visualization of Human Epidermal Receptor 2-overexpressed Gastric Cancer. J. Gastric Cancer.

[12] Boerner T., Piso P. (2021). Cytoreductive Surgery for Peritoneal Carcinomatosis from Gastric Cancer: Technical Details. J. Clin. Med..

[13] Wei J., Bu Z. (2020). Sentinel lymph node detection for gastric cancer: Promise or pitfall?. Surg. Oncol..

[14] Ikoma N., Blum M., Chiang Y.-J., Estrella J.S., Roy-Chowdhuri S., Fournier K., Mansfield P., Ajani J.A., Badgwell B.D. (2016). Yield of staging laparoscopy and lavage cytology for radiologically occult peritoneal carcinomatosis of gastric cancer. Ann. Surg. Oncol..

[15] Yao K., Doyama H., Gotoda T., Ishikawa H., Nagahama T., Yokoi C., Oda I., Machida H., Uchita K., Tabuchi M. (2014). Diagnostic performance and limitations of magnifying narrow-band imaging in screening endoscopy of early gastric cancer: A prospective multicenter feasibility study. Gastric Cancer.

[16] Kim Y.W., Min J.S., Yoon H.M., An J.Y., Eom B.W., Hur H., Lee Y.J., Cho G.S., Park Y.K., Jung M.R. (2022). Laparoscopic Sentinel Node Navigation Surgery for Stomach Preservation in Patients With Early Gastric Cancer: A Randomized Clinical Trial. J. Clin. Oncol..

[17] Kong S.-H., Noh Y.-W., Suh Y.-S., Park H.S., Lee H.-J., Kang K.W., Kim H.C., Lim Y.T., Yang H.-K. (2015). Evaluation of the novel near-infrared fluorescence tracers pullulan polymer nanogel and indocyanine green/γ-glutamic acid complex for sentinel lymph node navigation surgery in large animal models. Gastric Cancer.

[18] Van Driel P.B., van der Vorst J.R., Verbeek F.P., Oliveira S., Snoeks T.J., Keereweer S., Chan B., Boonstra M., Frangioni J., Van Bergen En Henegouwen P. (2014). Intraoperative fluorescence delineation of head and neck cancer with a fluorescent anti-epidermal growth factor receptor nanobody. Int. J. Cancer.

[19] Reja S.I., Minoshima M., Hori Y., Kikuchi K. (2021). Near-infrared fluorescent probes: A next-generation tool for protein-labeling applications. Chem. Sci..

[20] Shafirstein G., Bäumler W., Hennings L.J., Siegel E.R., Friedman R., Moreno M.A., Webber J., Jackson C., Griffin R.J. (2012). Indocyanine green enhanced near-infrared laser treatment of murine mammary carcinoma. Int. J. Cancer.

[21] Belia F., Biondi A., Agnes A., Santocchi P., Laurino A., Lorenzon L., Pezzuto R., Tirelli F., Ferri L., D’Ugo D. (2022). The use of indocyanine green (ICG) and near-infrared (NIR) fluorescence-guided imaging in gastric cancer surgery: A narrative review. Front. Surg..

[22] Nagaya T., Nakamura Y.A., Choyke P.L., Kobayashi H. (2017). Fluorescence-Guided Surgery. Front. Oncol..

[23] Gioux S., Choi H.S., Frangioni J.V. (2010). Image-guided surgery using invisible near-infrared light: Fundamentals of clinical translation. Mol. Imaging.

[24] Boogerd L.S.F., Hoogstins C.E.S., Schaap D.P., Kusters M., Handgraaf H.J.M., van der Valk M.J.M., Hilling D.E., Holman F.A., Peeters K., Mieog J.S.D. (2018). Safety and effectiveness of SGM-101, a fluorescent antibody targeting carcinoembryonic antigen, for intraoperative detection of colorectal cancer: A dose-escalation pilot study. Lancet Gastroenterol. Hepatol..

[25] Hoogstins C.E., Boogerd L.S., Sibinga Mulder B.G., Mieog J.S.D., Swijnenburg R.J., van de Velde C.J., Farina Sarasqueta A., Bonsing B.A., Framery B., Pèlegrin A. (2018). Image-guided surgery in patients with pancreatic cancer: First results of a clinical trial using SGM-101, a novel carcinoembryonic antigen-targeting, near-infrared fluorescent agent. Ann. Surg. Oncol..

[26] Framery B., Gutowski M., Dumas K., Evrard A., Muller N., Dubois V., Quinonero J., Scherninski F., Pelegrin A., Cailler F. (2019). Toxicity and pharmacokinetic profile of SGM-101, a fluorescent anti-CEA chimeric antibody for fluorescence imaging of tumors in patients. Toxicol. Rep..

[27] Warmerdam M.I., Creemers D.M.J., Kusters M., Peeters K., Holman F.A., Mieog J.S.D., Cailler F., Burger P., Burggraaf J., Rutten H.J.T. (2025). Long-term Local Control Following CEA-targeted Fluorescence-guided Surgery in Patients With Locally Advanced and Recurrent Rectal Cancer. Mol. Imaging Biol..

[28] Yim J.J., Harmsen S., Flisikowski K., Flisikowska T., Namkoong H., Garland M., van den Berg N.S., Vilches-Moure J.G., Schnieke A., Saur D. (2021). A protease-activated, near-infrared fluorescent probe for early endoscopic detection of premalignant gastrointestinal lesions. Proc. Natl. Acad. Sci. USA.

[29] Jang B., Lee S.H., Dovirak I., Kim H., Srivastava S., Teh M., Yeoh K.G., So J.B., Tsao S.K.K., Khor C.J. (2024). CEACAM5 and TROP2 define metaplastic and dysplastic transitions in human antral gastric precancerous lesions and tumors. Gastric Cancer.

[30] Luan F., Xu S., Chen K., Chen K., Kang M., Chen G., Chen J. (2025). Prognostic effect of CEA, AFP, CA19-9 and CA242 for recurrence/metastasis of gastric cancer following radical gastrectomy. Mol. Clin. Oncol..

[31] Han S.U., Kwak T.H., Her K.H., Cho Y.H., Choi C., Lee H.J., Hong S., Park Y.S., Kim Y.S., Kim T.A. (2008). CEACAM5 and CEACAM6 are major target genes for Smad3-mediated TGF-beta signaling. Oncogene.

[32] Uhlén M., Fagerberg L., Hallström B.M., Lindskog C., Oksvold P., Mardinoglu A., Sivertsson Å., Kampf C., Sjöstedt E., Asplund A. (2015). Tissue-based map of the human proteome. Science.

[33] de Gouw D., Rijpkema M., de Bitter T.J.J., Baart V.M., Sier C.F.M., Hernot S., van Dam G.M., Nagtegaal I.D., Klarenbeek B.R., Rosman C. (2020). Identifying Biomarkers in Lymph Node Metastases of Esophageal Adenocarcinoma for Tumor-Targeted Imaging. Mol. Diagn. Ther..

[34] Cox K.E., Amirfakhri S., Lwin T.M., Hosseini M., Ghosh P., Obonyo M., Hoffman R.M., Yazaki P.J., Bouvet M. (2025). A new locoregional mouse model of gastric cancer for identifying probes for fluorescence guided surgery. Surgery.

[35] Cristaudo A.T., Morris D.L. (2025). Prognostic value of carcinoembryonic antigen in colorectal adenocarcinoma: Expanding hypotheses into clinical practice. Clin. Exp. Med..

[36] Houvast R.D., van Duijvenvoorde M., Thijse K., de Steur W.O., de Geus-Oei L.F., Crobach A., Burggraaf J., Vahrmeijer A.L., Kuppen P.J.K. (2025). Selecting Targets for Molecular Imaging of Gastric Cancer: An Immunohistochemical Evaluation. Mol. Diagn. Ther..

[37] Huang S.C., Chang S.C., Liao T.T., Yang M.H. (2024). Detection and clinical significance of CEACAM5 methylation in colorectal cancer patients. Cancer Sci..

[38] Lin E., Lo Y.C., Subbiah V., Thawani R., Desai A. (2025). Advancing precision antibody-drug conjugate therapy: Unique proteogenomic profiles of tumor subsets in non-small cell lung cancer. Exp. Hematol. Oncol..

[39] Carvalho E., Canberk S., Schmitt F., Vale N. (2025). Molecular Subtypes and Mechanisms of Breast Cancer: Precision Medicine Approaches for Targeted Therapies. Cancers.

[40] Hatano T., Tanei T., Seno S., Sota Y., Masunaga N., Mishima C., Tsukabe M., Yoshinami T., Miyake T., Shimoda M. (2025). High HER2 Intratumoral Heterogeneity Is Resistant to Anti-HER2 Neoadjuvant Chemotherapy in Early Stage and Locally Advanced HER2-Positive Breast Cancer. Cancers.

[41] Nishino H., Turner M.A., Amirfakhri S., Hollandsworth H.M., Lwin T.M., Hosseini M., Framery B., Cailler F., Pèlegrin A., Hoffman R.M. (2022). Proof of concept of improved fluorescence-guided surgery of colon cancer liver metastasis using color-coded imaging of a tumor-labeling fluorescent antibody and indocyanine green restricted to the adjacent liver segment. Surgery.

[42] Schaap D., Valk K., Deken M., Meijer R., Burggraaf J., Vahrmeijer A., Kusters M., Kusters M., Boogerd L., Schaap D. (2020). Carcinoembryonic antigen-specific, fluorescent image-guided cytoreductive surgery with hyperthermic intraperitoneal chemotherapy for metastatic colorectal cancer. J. Br. Surg..

[43] Tiernan J., Perry S., Verghese E., West N., Yeluri S., Jayne D., Hughes T. (2013). Carcinoembryonic antigen is the preferred biomarker for in vivo colorectal cancer targeting. Br. J. Cancer.

[44] de Valk K.S., Deken M.M., Schaap D.P., Meijer R.P., Boogerd L.S., Hoogstins C.E., van der Valk M.J., Kamerling I.M., Bhairosingh S.S., Framery B. (2021). Dose-finding study of a CEA-targeting agent, SGM-101, for intraoperative fluorescence imaging of colorectal cancer. Ann. Surg. Oncol..

[45] Gutowski M., Framery B., Boonstra M.C., Garambois V., Quenet F., Dumas K., Scherninski F., Cailler F., Vahrmeijer A.L., Pelegrin A. (2017). SGM-101: An innovative near-infrared dye-antibody conjugate that targets CEA for fluorescence-guided surgery. Surg. Oncol..

[46] Yanagihara K., Takigahira M., Mihara K., Kubo T., Morimoto C., Morita Y., Terawaki K., Uezono Y., Seyama T. (2013). Inhibitory effects of isoflavones on tumor growth and cachexia in newly established cachectic mouse models carrying human stomach cancers. Nutr. Cancer.

[47] Chou Y.J., Chang C.L., Tsai Y.C. (2025). Diagnostic Accuracy of Indocyanine Green-stained Sentinel Lymph Nodes in Prostate Cancer Patients: A Systematic Review and Meta-analysis. Eur. Urol. Open Sci..

[48] Kalayarasan R., Chandrasekar M., Sai Krishna P., Shanmugam D. (2023). Indocyanine green fluorescence in gastrointestinal surgery: Appraisal of current evidence. World J. Gastrointest. Surg..

[49] Tichauer K.M., Deharvengt S.J., Samkoe K.S., Gunn J.R., Bosenberg M.W., Turk M.J., Hasan T., Stan R.V., Pogue B.W. (2014). Tumor endothelial marker imaging in melanomas using dual-tracer fluorescence molecular imaging. Mol. Imaging Biol..

[50] van Gennep E.J., Pisano G., KleinJan G.H., Rietbergen D.D.D., Hendricksen K., Mertens L.S., Vd Kamp M.W., Wit E.M.K., van Montfoort M.L., Donswijk M. (2025). Prospective clinical study of sentinel node detection in bladder cancer using a hybrid tracer—Towards replacement of pelvic lymph node dissection in cases with sentinel node visualization on SPECT/CT?. Eur. J. Nucl. Med. Mol. Imaging.

[51] Wu X., Feng S., Chang T.S., Zhang R., Jaiswal S., Choi E.K., Duan Y., Jiang H., Wang T.D. (2024). Detection of Hepatocellular Carcinoma in an Orthotopic Patient-Derived Xenograft with an Epithelial Cell Adhesion Molecule-Specific Peptide. Cancers.

[52] Zonoobi E., Neijenhuis L.K.A., Lemij A.A., Linders D.G.J., Nazemalhosseini-Mojarad E., Bhairosingh S.S., Dekker-Ensink N.G., van Vlierberghe R.L.P., Peeters K., Holman F.A. (2025). Impact of Neoadjuvant Treatment on Target Expression in Rectal Cancer for Near-Infrared Tumor Imaging. Cancers.

[53] Boonstra M.C., De Geus S.W., Prevoo H.A., Hawinkels L.J., Van De Velde C.J., Kuppen P.J., Vahrmeijer A.L., Sier C.F. (2016). Selecting targets for tumor imaging: An overview of cancer-associated membrane proteins. Biomark. Cancer.

[54] Jansen K., Kornfeld L., Lennartz M., Dwertmann Rico S., Kind S., Reiswich V., Viehweger F., Bawahab A.A., Fraune C., Gorbokon N. (2024). Carcinoembryonic Antigen Expression in Human Tumors: A Tissue Microarray Study on 13,725 Tumors. Cancers.

[55] Beauchemin N., Arabzadeh A. (2013). Carcinoembryonic antigen-related cell adhesion molecules (CEACAMs) in cancer progression and metastasis. Cancer Metastasis Rev..

[56] Croce A.C., Bottiroli G. (2014). Autofluorescence spectroscopy and imaging: A tool for biomedical research and diagnosis. Eur. J. Histochem..

[57] Baugh L.M., Liu Z., Quinn K.P., Osseiran S., Evans C.L., Huggins G.S., Hinds P.W., Black L.D., Georgakoudi I. (2017). Non-destructive two-photon excited fluorescence imaging identifies early nodules in calcific aortic-valve disease. Nat. Biomed. Eng..

[58] Cowles E.A., Kovar J.L., Curtis E.T., Xu H., Othman S.F. (2013). Near-infrared optical imaging for monitoring the regeneration of osteogenic tissue-engineered constructs. Biores Open Access.

[59] Ohuchi N., Wunderlich D., Fujita J., Colcher D., Muraro R., Nose M., Schlom J. (1987). Differential expression of carcinoembryonic antigen in early gastric adenocarcinomas versus benign gastric lesions defined by monoclonal antibodies reactive with restricted antigen epitopes. Cancer Res..

[60] Batra P., Narasannaiah A.H., Reddy V., Subramaniyan V., Manjunath K.V., Yeshwanth R., Arjunan R., Althaf S., Chunduri S., Anwar A.Z. (2023). Prognostic Value of Tumor Markers in Gastric Cancer: A Tertiary Cancer Centre Experience. Cureus.

[61] Huang Z., Zhao X., Hu J., Zhang C., Xie X., Liu R., Lv Y. (2022). Single-Nanoparticle Differential Immunoassay for Multiplexed Gastric Cancer Biomarker Monitoring. Anal. Chem..

[62] Zhou J. (2025). Challenges and perspectives of CAR-T cell therapy in solid tumours: Insights from gastric cancer. Br. J. Cancer.

