# Supplementary material for: Feasibility of Anti-CEA Dye Conjugate for Cancer-Specific Imaging in Gastric Cancer Cell Lines and Mouse Xenograft Models
Source: Cancers (Basel). 2025 Sep 8;17(17):2937. doi: 10.3390/cancers17172937 (PMC12428090; doi:10.3390/cancers17172937)
Supplement: Supplementary file 1 [file cancers-17-02937-s001.zip › cancers-3824367-supplementary.pdf]

Supplementary Materials

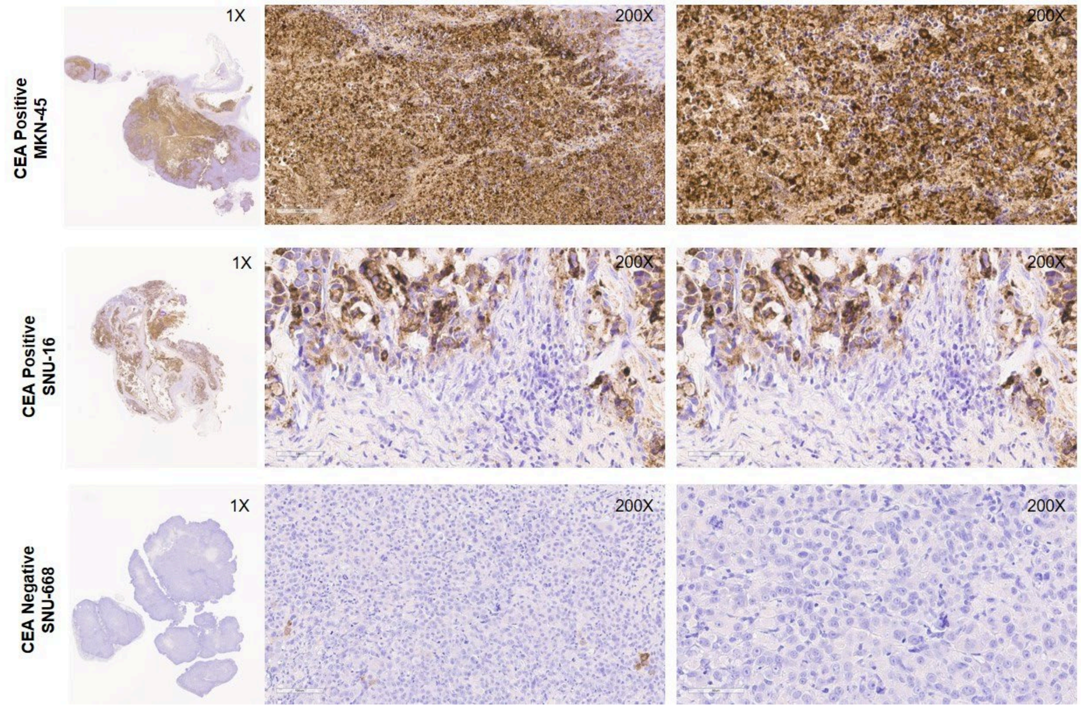

Supplementary Figure S1 Ex vivo tumor histological image. All slides were confirmed by a pathologist using Hematoxylin and Eosin staining. The left panel shows a full scan view image of the tumor sample, providing an overview of the entire sample. MKN-45, SNU-16, SNU-668. The middle panel displays a magnified view (200X) of the region of interest, the last panel displays a magnified view(400X) of the region of interest, highlighting the specific features of morphology.

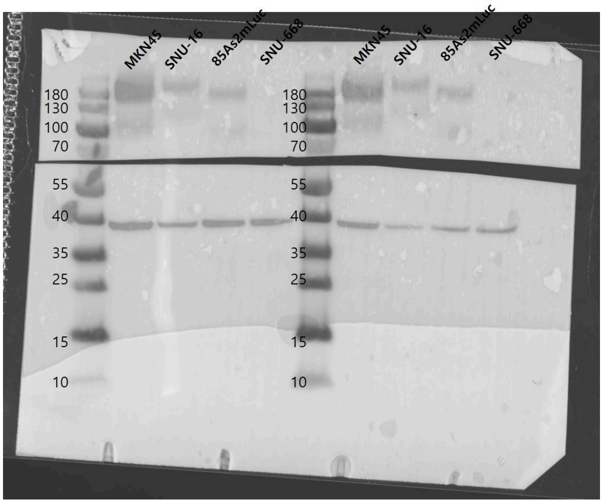

Supplementary Figure S2 original images of western blot
